# Altered immune parameters associated with Koala Retrovirus (KoRV) and Chlamydial infection in free ranging Victorian koalas (*Phascolarctos cinereus*)

**DOI:** 10.1038/s41598-019-47666-8

**Published:** 2019-08-01

**Authors:** Iona E. Maher, Jade Patterson, Megan Curnick, Joanne Devlin, Damien P. Higgins

**Affiliations:** 1 Sydney School of Veterinary Science, Faculty of Science, The University of Sydney, Sydney, NSW Australia; 2 Asia Pacific Centre for Animal Health, Melbourne Veterinary School, Faculty of Veterinary and Agricultural Sciences, The University of Melbourne, Melbourne, VIC Australia; 3 Veterinary Department, Melbourne Zoo, Parkville, VIC Australia

**Keywords:** Infectious diseases, Zoology

## Abstract

Koala Retrovirus (KoRV) has been widely speculated to cause immune suppression in koalas (*Phascolarctos cinereus*) and to underlie the koala’s susceptibility to infectious disease, however evidence for immunomodulation is limited. The aim of this study is to determine whether immunophenotypic changes are associated with KoRV infection in free ranging Victorian koalas. qPCR was used to examine mRNA expression for Th1 (IFNγ), Th2-promoting (IL6, IL10) and Th17 (IL17A) cytokines, along with CD4 and CD8 in whole blood of koalas (n = 74) from Mt Eccles and Raymond Island in Victoria, Australia, with and without natural chlamydial infection. KoRV positive koalas had significantly lower levels of IL17A (*p*`*0*.*023*) and IFNγ (*p* = *0*.*044*) gene expression along with a decreased CD4:CD8 gene expression ratio (*p* = *0*.*025*) compared to negative koalas. No effect of chlamydial infection or combined effect of KoRV and chlamydial infection was detected in these populations. The decreased expression of IFNγ could make KoRV infected koalas more susceptible to persistent chlamydial infection, and a decrease in IL17A could make them more susceptible to gram negative bacterial, fungal and mycobacterial infection; but more tolerant of chlamydial infection.

## Introduction

Koala Retrovirus (KoRV) is a gamma retrovirus that has been widely speculated to cause immune suppression in koalas (*Phascolarctos cinereus*) and to underlie the koala’s susceptibility to infectious disease. The variant KoRV A, is endogenous and at 100% prevalence in the northern majority of the koala’s range (the states of Queensland and New South Wales (NSW), where the koala is listed as Vulnerable)^1^. Although it is endogenous, it has open reading frames, is actively transcribed and all koalas are viraemic^2,3^. KoRV A is exogenous in the southern states of Victoria and South Australia, with variable prevalence^1,2,4^ and a much lower proviral number per cell^1^. It has been postulated that this is an exogenous retrovirus that is currently in the process of endogenizing^2^. KoRV A is the only subtype so far detected in Victoria^4^.

Evidence for an effect of KoRV on the immune system of koalas is limited and is largely based on the argument that the spectrum of diseases that northern koalas frequently suffer from, which include neoplasia (e.g. lymphoma, leukaemia, mesothelial and craniofacial tumours) and infectious diseases (e.g. *Chlamydia*, cryptococcosis, and a range of opportunistic infections), is similar to that seen in cats infected by the closely related gamma-retrovirus, feline leukaemia virus (FeLV)^5,6^. The role of KoRV as an immune modulator has previously been questioned as most koalas infected with endogenous KoRV A are healthy, and authors have suggested co-factors may be necessary to cause disease^3^. Recent studies have, however, reported an association between exogenous KoRV infection and wet bottom (fur staining associated with chronic urinary incontinence) and low body condition score in Victorian koalas^4^ and an association between the exogenous subtype KoRV B, and chlamydial disease^7^ in Queensland koalas. Immunological studies have potential to further validate such observations and provide information on mechanisms involved.

Experimental evidence for an effect of KoRV A on the immune system is limited to a single study in which human peripheral blood mononuclear cells (PBMC’s) incubated with KoRV A increased expression of the Th2 associated cytokines Interleukin 6 (IL6) and Interleukin 10 (IL10)^8^. Such studies have not been performed on koala cells. KoRV does contain several structural elements that are associated with immune suppression by closely related gamma retroviruses such as FeLV and Murine Leukaemia Virus (MuLV). These include the viral transmembrane protein p15E^5^, which is associated with a multitude of immune suppressive effects including inhibition of lymphocyte activation by mitogens and modulation of cytokine expression by peripheral blood mononuclear cells (PBMC’s)^5^. CKS17, a 17 amino acid sequence that corresponds to a highly conserved portion of the hydrophilic region of p15E, is reported to down regulate production of Th1 cytokines, including Interferon gamma (IFNγ), Tumour Necrosis Factor alpha (TNFα), Interleukin 2 (IL2), and to up regulate Th1 inhibiting cytokines, including IL10, in rodents and humans (reviewed in^9^).

*Chlamydia* is considered to be the most important pathogen of koalas^10^ and control of chlamydial disease has been identified as the key component in long term survival of some threatened koala populations in NSW and Queensland^11,12^. Based on our current understanding of chlamydial pathogenesis, we may expect that inhibition of Th1 pathways would be associated with persistence and increased pathogenesis of chlamydial disease in infected koalas. The immune response to *Chlamydia* has been studied extensively in humans and animal models and it is established that elimination of chlamydial infection is reliant upon Th1 cytokines, such as IFNγ, which promote cytotoxic T cell responses^13^. The immunological hypothesis of chlamydial pathogenesis states that Th2 cells generated in response to infection with *Chlamydia* spp. may down regulate the protective Th1-type immune responses and promote persistent infection^14,15^. Supporting this, a Th2-dominated response, characterised by increased Interleukin 4 (IL4) and promoted by IL10, is associated with persistence of infection (reviewed in Menon *et al*.^16^). Although population wide studies in humans are limited, researchers have consistently found that IL10 promoting polymorphisms are associated with increased ocular scarring and infertility in people with ocular and genital chlamydial infections^17,18^.

Outside of the Th1-Th2 paradigm, the role of the Th17 immune profile appears to play an important role in chlamydial infection as the magnitude and duration of infection with *C*. *muridarum* in mice is significantly decreased in the absence of Interleukin 17 (IL17)^19^. In koalas, PBMC’s from those with chlamydial disease had greater gene expression for IL17A, but not IL10, IFNγ or TNFα, when stimulated with inactivated *Chlamydia pecorum*^20^, which may indicate that the Th17 response also plays an important role in immune-mediated chlamydial pathogenesis in that species.

Other factors proposed to influence the immune response of koalas include stress^21^, fluctuations in dietary plant secondary metabolites^22,23^, nutrient availability^24^, genetic differences^25,26^, sex^27^, hormonal fluctuations, season and photoperiod^28^. Subclinical infections with other organisms such as recently discovered Trypanosomes^29–32^, herpesviruses^33,34^ or unknown pathogens could also play a role. Our previous work indicates that season has a marked effect on cytokine expression and the CD4:CD8 expression ratio in koalas^35^.

A reduction in CD4:CD8 ratio is often used as a measure of progression of immunosuppression in individuals infected with retroviruses including HIV, FIV and, to a lesser extent, FeLV^36–40^. The impact of KoRV and chlamydial infection on the CD4:CD8 ratio in naturally infected free-ranging koalas is difficult to predict; in more closely related gammaretroviruses, such as most strains of FeLV, the ratio is often unchanged^41^. Our previous studies have shown that the CD4:CD8 mRNA ratio varies markedly with season in koalas but there was no significant difference in koalas infected with KoRV B and those not infected (all koalas were KoRV A positive)^35^.

Immunological studies of wild animals are uncommon and are inherently difficult due to the greater variation (environmental, genetic, nutritional, parasitic, hormonal, microbiological etc.) that is present, relative to the highly controlled models used in laboratory immunology. However, it is only in the context of these wild settings that we can measure the association of immune phenotypes with host health and fitness and the likely interplay of environment^42^. This is a novel and exploratory approach to the examination of the immune system and although they are gaining momentum, the fields of wild immunology and eco-immunology are informing the significant challenges of transferring laboratory concepts to free-ranging populations of animals and, indeed, humans^42^.

In this challenging context, the current study seeks to determine whether an association between KoRV infection and cytokine and CD4:CD8 gene expression can be detected in free-ranging koalas. We make use of natural infection by *Chlamydia spp*. to permit study of the immune system at rest and under a natural immune stimulus. We hypothesise that immune parameters will differ between KoRV positive and negative koalas, and *Chlamydia spp*. positive and negative koalas and that KoRV positive animals may respond differently to the immune challenge of chlamydial infection compared to KoRV negative animals. We examine resting IFNγ, IL6, IL10 and IL17A, CD4 and CD8 mRNA expression in koalas in two populations in Victoria (Mt Eccles and Raymond Island), in which KoRV A and *Chlamydia pecorum* infect koalas.

## Results

The sample population consisted of 74 koalas, 47 from Mt Eccles (all female) and 27 from Raymond Island (16 female, 11 male). Multivariate linear mixed model detected no significant effect of collection site on overall cytokine gene expression, thus the data from Mt Eccles and Raymond Island (which are at similar latitudes and were collected within one month of each other) were combined for analysis. The KoRV and *Chlamydia* status of the populations is presented in Table 1. Sixteen animals had dual KoRV and *C*. *pecorum* infections, 13 were KoRV negative and *C*. *pecorum* positive, 12 were KoRV positive and *C*. *pecorum* negative and 33 were KoRV and *C*. *pecorum* negative. Reference gene cytokine mRNA levels could be quantified in 74 samples and, of those, IL17A mRNA was quantifiable in 68 samples, IL10 in 61, IFNγ in 55, IL6 in 52, CD4 in 42, CD8 in 51 and CD4:CD8 in 39.

**Table 1:** Koala retrovirus (KoRV) and *Chlamydia pecorum* PCR status of the koalas sampled.

Site	KoRV + *C*. *pecorum* −	KoRV − *C*. *pecorum* +	KoRV − *C*. *pecorum* +	KoRV − *C*. *pecorum* −	Total
Mt Eccles	11	6	8	22	47
Raymond Island	5	7	4	11	27
Total	16	13	12	33	74

There was a trend for overall cytokine gene expression to be lower in KoRV-positive koalas (multivariate linear mixed model; *p* = *0*.*098*) and a significant effect of sex (multivariate linear mixed model; *p* = *0*.*008*) on overall cytokine gene expression with males being higher than females. No overall effects of other variables, including *Chlamydia* infection status, age, abnormal health status, presence of pouch or back young were found. There was no overall interaction between KoRV and *Chlamydia* infection. There was no significant association between sex and KoRV (χ^2^ test; *p* = *0*.*77*) or *Chlamydia* status (χ^2^ test; *p* = *0*.*55*).

**Figure 1:**
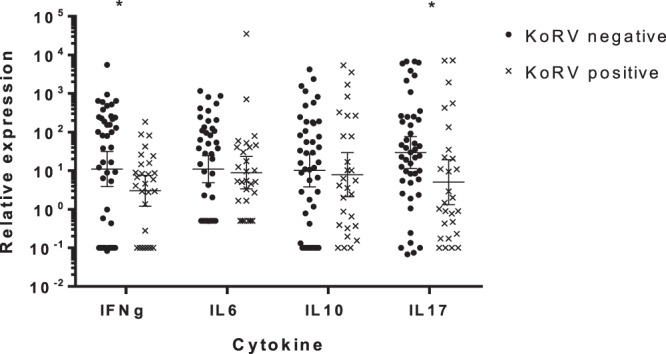
The effect of Koala retrovirus (KoRV) status on cytokine expression. Geometric mean and 95%CI of the geometric mean for relative expression of cytokine levels, compared to reference genes calculated using the ΔCq method^87^. IFNγ and IL17A were significantly decreased in KoRV positive koalas (*p < *0*.*05* Mann-Whitney U test).

**Figure 2:**
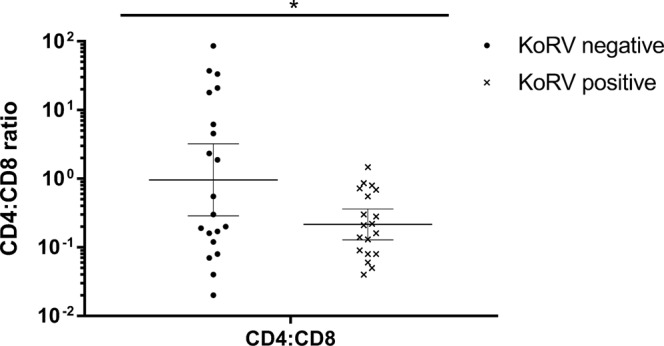
Effect of KoRV infection on CD4:CD8 expression ratio of KoRV negative (left, n = 20) vs KoRV infected (right, n = 19) koalas. Geometric mean (line) and 95% CI of the geometric mean for CD4:CD8 relative expression ratio calculated from CD gene expression relative to reference genes calculated using the ΔCq method^67^. The CD4:CD8 expression ratio is significantly lower in koala retrovirus (KoRV) positive koalas (*p < *0*.*05*, T test with Welch’s correction for uneven variance), and less variable.

**Figure 3:**
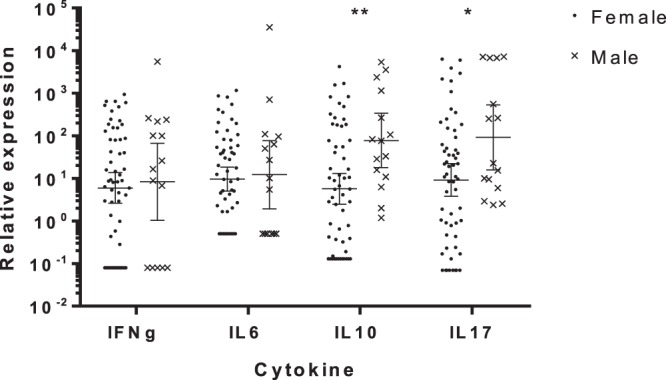
The effect of sex on cytokine expression. Geometric mean and 95%CI of the geometric mean for relative expression of cytokine levels, compared to reference genes calculated using the ΔCq method^67^. IL10 and IL17A were significantly greater in males (*p < *0*.*05*, **p < *0*.*01* Mann*-*Whitney U test).

When the expression of genes was analysed individually, IL17A (Mann-Whitney; *p* = *0*.*023*) and IFNγ (Mann-Whitney; *p* = *0*.*044*) gene expression (Fig. 1) and CD4:CD8 gene expression ratio (t test; *p* = *0*.*025*; Fig. 2) were significantly lower in KoRV positive koalas compared to negative koalas. Males had significantly higher resting expression of IL10 (Mann-Whitney; *p* = *0*.*006*) than females. As males also had significantly higher resting expression of IL17A (Mann-Whitney; *p* = *0*.*03*) (Fig. 3) analysis of KoRV effect on IL17 was repeated for females only and remained significant (Mann-Whitney; *p* = *0*.*04*). There was no apparent effect of the presence of pouch or back young, abnormal health status or age on expression of any individual cytokine or on CD4:CD8 expression ratio.

There was a non-significant trend for an association between KoRV and chlamydial infection, as 68% of KoRV positive animals were also positive for *Chlamydia* compared with 42% of KoRV negative animals (χ^2^ test *p* = *0*.*19*). There was no effect of *Chlamydia* infection status or any additional effect of dual infection with KoRV and *Chlamydia* infection on cytokine expression.

## Discussion

This is the first report of immunophenotypic changes associated with KoRV infection and sex in free-ranging koalas. Although this study is unable to determine the biological impact of the changes observed, the lower resting expression of IFNγ, IL17A genes and lower CD4:CD8 gene expression ratio in KoRV-infected koalas provides a way forward to further explore mechanisms behind recently reported association of KoRV infection with disease in these populations^4^. To the authors’ knowledge this is the first study of this type in koalas or marsupials in general and demonstrates that, although studies on wildlife immunology contain the inherent risks that potential variation caused by the disease of interest will be undetectable, this approach can provide valuable information about the immune response and infectious diseases in koalas and wildlife in general within the context of their normal environment. Sex-effects on cytokine gene expression will be important to consider in future immunological studies of koalas.

Studies examining the effect of infectious disease on cytokine expression in wild or semi wild animals are limited, as immunological methods are usually species specific. Most studies are recent and, similar to this study, use real time PCR for cytokine mRNA quantification. Even with the high sensitivity of these assays cytokine mRNA is often below quantifiable limits in non-stimulated cell samples^43^ and some studies have found mostly inconclusive results^44^. The study of wildlife immunology is in its infancy and transfer of our knowledge of immunology, which is mostly derived from laboratory animals, to wild-living animals will be difficult but is expected to develop as technological (species specific reagents and annotated genomes) and statistical methods improve^42^. Koala populations are coming under increasing threat, especially in the northern part of their range, and studies such as this are an essential component of understanding the immune mechanisms underpinning this susceptibility to infectious disease and to help these threatened populations survive.

The present study has produced several findings that indicate follow-up flow cytometric studies using novel or cross-reactive antibodies to CD4^45^, IFNγ^46^ or IL17 would be worthwhile. The decision to use baseline gene expression in the present study, rather than cells preserved for flow cytometry, was driven by field constraints on sample storage and has its limitations; it will be important to determine whether reduced CD4:CD8 gene expression ratio and reduced expression of IFNγ and IL17 genes in the present study is related to gene down-regulation, or deletion of cells. Depletion of CD4+ cells has been reported with some fatal strains of the gammaretrovirus FeLV^38,40^ and Th1 lymphocyte depletion can be induced by HIV^47,48^. The lower level of IFNγ gene expression observed in KoRV infected koalas in our study is suggestive of a switch from Th1 to Th2 CD4+ cell activity but this could not be directly examined as IL4, a key Th2 cytokine, was below quantifiable levels in our study.

It may be that KoRV infection is similar in pathogenesis to feline retroviral infections (FIV and FeLV), which are associated with a general cytokine dysregulation^49–51^ rather than a clear Th1 to Th2 switch. The decrease seen in the CD4:CD8 gene expression ratio and IL17 and IFNγ gene expression in KoRV infected koalas is consistent with the effect of other retroviruses (HIV, FIV and to a lesser degree FeLV) in humans and felids^36–40^ but the pattern is not clear cut in that, although the changes observed would be consistent with the up-regulation of IL10 observed in human lymphocytes incubated with KoRV *in-vitro*^8^, no significant difference in IL10 expression was seen in association with KoRV in the present study.

Functional studies involving lymphocyte stimulation are also likely to add a valuable additional perspective. Human studies looking at both unstimulated and stimulated blood cytokine levels have found contrasting values (e.g. lower unstimulated but higher mitogen stimulated expression of IL6 and TNFα in diabetic patients compared to controls^52^). Additionally, our previous study that examined cytokine expression among KoRV A/B positive and KoRV A positive animals found KoRV B infection was associated with increased up-regulation of all measured cytokines (IFNγ, TNFα, IL4, IL6, IL10 and IL17A) in stimulated cells but in unstimulated cells a difference was only detected in IL10 gene expression^35^.

The changes seen in association with KoRV infection in the current study are potentially relevant to the recent detection of an association between wet bottom (rump staining by urine, which is most commonly associated with chronic chlamydial disease) and KoRV infection in Victorian koalas^4^. As CD4+ cells play an essential role in the control of chlamydial infection in both humans and the murine model^46,53^, depletion of CD4+ cells or reduction in CD4 expression could also play a role in making KoRV infected koalas more susceptible to chlamydial infection and disease. The lower levels of IFNγ expression associated with KoRV infection in this study could also, in theory, make koalas more susceptible to chlamydial persistence, as IFNγ production is essential to eliminate or prevent the dissemination of infection^13^. Blocking production of IFNγ significantly prolongs chlamydial infection^54^, exacerbates pathological lesions and increases chlamydial load^55^. There is also evidence in humans that haplotypes associated with reduced IFNγ production are associated with increased scarring secondary to ocular chlamydial infections^56^. If KoRV infected koalas have a reduced IFNγ response when exposed to chlamydial infection this could result in increased dissemination, higher pathogen burden, prolongation of disease and increase in pathological lesions in these animals. The relevance of reduced Th17 expression to chlamydial disease in koalas requires further investigation; while it is likely an important component of defence, elevated Th17 responses are also associated with chlamydial pathology^19^ so limiting Th17 responses may either exacerbate pathogenesis by prolonging infection or moderate immune-mediated pathogenesis.

Although there was a tendency for association between KoRV and chlamydial infection in this cohort it was not statistically significant and there was no relationship between chlamydial infection and cytokine expression or CD4:CD8 expression ratio. This may be related to the inability of the chlamydial PCR assay to discriminate between animals with clinical and subclinical disease, which would be expected to have differing immunophenotypes. Similar to other southern Australian populations^10,57,58^, the populations studied have high levels of sub-clinical chlamydial infection^59^.

The effect of sex on immune parameters in the current study is a vital consideration for future immunological studies in this species. The greater expression of IL10 and IL17A genes in males, relative to females, is likely to reflect that, unsurprisingly, sex hormones influence the resting expression of these cytokines in koalas. These samples were taken during the koala mating season^60^, and the greater IL10 expression in males may be associated with increased testosterone production, as testosterone is associated with increased IL10 production in humans and mice^61,62^. The sex hormone effect on IL17 production has been less well studied, however oestrogen has been found to inhibit Th17 cell differentiation in mice^63^ and might explain the lower levels of IL17 in found in female koalas in this study.

## Conclusion

This study presents the first evidence that exogenous KoRV A infection is associated with alterations in the immune system of koalas. Despite the heterogeneity of free ranging animals, a statistically significant reduction in resting IFNγ and IL17A levels and decrease in the CD4:CD8 gene expression ratio was found in koalas infected with KoRV A. The decreased expression of IFNγ could potentially make KoRV infected koalas more susceptible to disseminated chlamydial disease and could be an underlying mechanism behind the recently discovered association between KoRV infection and wet bottom in Victorian koalas^4^; however the current study does not confirm a role for KoRV in disease but rather reports associative relationships between KoRV infection and immune parameters that will be useful in targeting future *in vitro* and functional studies of causation and pathogenesis. Such studies would include examination of the cytokine response of koala cells (e.g. PBMC’s) or cell lines to KoRV infection *in-vitro*, along with characterisation of the CD4+ and CD8+ cell responses in flow cytometric studies. It comprises a first step on the path to understanding the immune mechanisms underpinning the susceptibility to infectious disease in koala populations, which is essential to help these threatened populations survive. It also demonstrates that, while inherently risky and challenging, wildlife immunology studies can provide valuable information about diseases and immune responses in wild animals.

## Materials and Methods

### Ethics statement

All animal handling and sampling was conducted under a permit issued by the Victorian Department of Sustainability and Environment and Parks Victoria (permit no. 10005388). The University of Melbourne Animal Ethics Committee granted approval for physical examinations and sample collection on animals that were captured and restrained for routine management operations (ethics 1011687.1).

### Koala sampling and clinical examination

The clinical examinations and sample collection for these koalas is described previously^59^. This occurred during routine population management operations conducted by the Victorian Department of Sustainability and Environment and Parks Victoria at Raymond Island (37.9088°S, 147.7545°E; October 2010) and Mt Eccles National Park (38.0667°S, 141.9167°E; September and October 2010).

Briefly, clinical examination included estimation of age from tooth wear, abdominal and lymph node palpation, scoring of “wet bottom” lesions (staining and maceration of rump epithelium and pelage), ocular or urogenital abnormalities, and body condition, along with noting the presence of pouch or back young. Teeth were graded as per the method of Martin^60^ and McLean^64^; age class was defined as young (0–3.5 years, tooth wear class 0–1), mature (3.5–10 years, tooth wear class 2–7) or old (>10 years, tooth wear class 8–9). Ultrasound examination was performed on animals that were anesthetized and did not have pouch young (n = 22, all female). The wall and lumen of uteri, the urinary bladder, and the width of each kidney were measured. Any other urogenital abnormalities, including para-ovarian cysts, were also noted. The data from the physical examination were used to classify the animals as being in normal or abnormal health; abnormal health was based on palpation of reduced gut fill, enlarged lymph nodes, reduced body condition, the presence of wet bottom or any ocular, urogenital abnormalities or ultrasonographic abnormalities.

A minimum of 2 mL of blood was collected from the cephalic vein into EDTA anticoagulant. Following centrifugation, plasma was withdrawn and 0.5 mL buffy coat was collected from each sample using a micropipette and stored in 1 mL RNAlater (Sigma-Aldrich, St Louis, MO, USA) for cytokine mRNA analysis. Remaining sample was frozen at −20 °C and then at −70 °C for longer-term storage for KoRV testing.

### KoRV and *chlamydia* testing

For KoRV provirus testing, DNA was extracted from 200 μL of each sample using DX Universal Liquid Sample DNA Extraction Kit (Qiagen, Doncaster, Vic, Australia) and a QIAextractor robot (Qiagen, Doncaster, Vic, Australia). Each 96 well plate had six negative control wells containing sterile water and one positive extraction control well containing diluted liquid culture of *E*. *coli* containing a portion of KoRV *pol* inserted into pGEM^®^-T (Promega) plasmid. Conventional PCR was used to screen clinical samples for the presence of KoRV proviral DNA. This PCR utilised primers targeting KoRV *pol* gene (5′TTGGAGGAGGAATACCGATTACAC- 3′ (sense) and 5′-GCCAGTCCCATACCTGCCTT-3′ (antisense))^65^ and thus amplified all KoRV variants though, to date, no variant other than KoRV A has been detected in Victorian koalas^4^. qPCR was performed using a GoTaq Flexi DNA polymerase PCR kit (Promega, Madison, WI, USA). Each 25 μL reaction contained 5 μL of GoTaq Flexi Buffer, 10 mM of MgCl, 125 μM of each dNTP, 50 μM of each primer, 1.5 U of GoTaq DNA Polymerase, 6.7 μL of RNase-free treated water and 5 μL of template DNA. Cycling conditions (Icycler Thermal 45 Cycler, Bio-Rad, Hercules, CA, USA) were 95 °C for 1 min, followed by 35 cycles of 95 °C for 30 sec, 53 °C for 30 sec, 72 °C for 1 min. Each PCR included a positive control (diluted purified cloned KoRV *pol* DNA) and negative control (water). Following PCR amplification, all samples were subjected to agarose gel electrophoresis. To confirm specific amplification of KoRV DNA, band excision, gel DNA extraction using QIAquick gel extraction kit (Qiagen, Doncaster, Vic, Australia), and nucleotide sequencing (BDT3.1 Chemistry, Applied Genetic Diagnostics, Department of Pathology, The University of Melbourne) was performed on products of the expected size (110 bp).

Sampling and testing for *Chlamydia*, on ocular and urogenital/penile swabs, by qPCR was performed as previously described^59^. For positive samples, chlamydial species was determined by qPCR HRM curve analysis; only *C*. *pecorum* was identified^59^.

### Cytokine expression analysis

The mRNA was extracted from buffy coat in RNAlater using the RNAeasy minikit (Qiagen, Doncaster, Vic, Australia) following manufacturer’s instructions. Extracted samples were treated with amplification grade DNAse 1 (AMPDI-1KT; Sigma Aldrich, MO, USA) to remove contaminating DNA, then cDNA was synthesised using the Revertaid first strand cDNA synthesis kit (Thermo Scientific, Lithuania). To control for contamination with genomic DNA ‘no reverse transcriptase’ (NRT) controls were made using the same protocol and omitting reverse transcriptase. The concentration and purity of RNA was assessed using a NanoDrop ND-1000 Spectrophotometer (Thermo Scientific, Wilmington, DE, USA).

Reference gene (28 s and GAPDH) and cytokine/CD (IL6, IL10, IFNγ, CD4 and CD8) qPCR were performed in triplicate as described previously^66^. Conditions for IL17A qPCR^20^ were re-optimised for our laboratory. Samples were excluded from analysis if the reference gene amplification fell below the limit of quantification^66^.

### Statistical analysis

Raw Cq values were transformed to 2^−*Cq*^ for analysis. Resting immune gene expression levels in samples were measured using the 2^−ΔCq^ method^67^. Cytokine expression was normalised against the geometric mean of the two reference genes 28 s and GAPDH. These have previously been found to be valid reference genes for koala cells^66^.

The data were log transformed and then Shapiro-Wilk analysis was used to confirm normality and a χ^2^ test used to confirm independence between sex, KoRV and chlamydial infection. Multivariate linear mixed models were then used to examine interactions across multiple cytokines and two-way ANOVA was used to examine interactions between cytokine expression and multiple independent variables (e.g. KoRV, *Chlamydia* status, or sex) (GenStat 17^th^ edition, VSN International Ltd.). Cytokine results below quantifiable limits were included in calculations at a value equal to the lowest measurable value, to avoid bias associated with exclusion of results below quantifiable limits. Therefore, a conservative non-parametric approach was taken when conducting post-hoc analysis: post hoc *Mann-Whitney U t*ests (GraphPad Prism 7.02) were performed, and only on cytokines that appeared most likely to differ between two populations (e.g. KoRV positive and negative) based on visual examination of scatterplot data. A *p* value of <0.05 was considered significant.

## References

[1] Simmons G (2012). Prevalence of koala retrovirus in geographically diverse populations in Australia. Australian Veterinary Journal.

[2] Tarlinton RE, Meers J, Young PR (2006). Retroviral invasion of the koala genome. Nature.

[3] Hanger JJ, Bromham LD, McKee JJ, O’Brien TM, Robinson WF (2000). The nucleotide sequence of koala (*Phascolarctos cinereus*) retrovirus: a novel type C endogenous virus related to Gibbon ape leukemia virus. Journal of Virology.

[4] Legione AR (2017). Koala retrovirus genotyping analyses reveal a low prevalence of KoRV-A in Victorian koalas and an association with clinical disease. Journal of Medical Microbiology.

[5] Denner J, Young PR (2013). Koala retroviruses: characterization and impact on the life of koalas. Retrovirology.

[6] Kinney ME, Pye GW (2016). Koala Retrovirus: a Review. Journal of Zoo and Wildlife Medicine.

[7] Waugh CA (2017). Infection with koala retrovirus subgroup B (KoRV-B), but not KoRV-A, is associated with chlamydial disease in free-ranging koalas (Phascolarctos cinereus). Scientific Reports.

[8] Fiebig U, Hartmann MG, Bannert N, Kurth R, Denner J (2006). Transspecies transmission of the endogenous koala retrovirus. Journal of Virology.

[9] Haraguchi S, Good RA, Day-Good NK (2008). A potent immunosuppressive retroviral peptide: cytokine patterns and signaling pathways. Immunologic Research.

[10] Polkinghorne A, Hanger J, Timms P (2013). Recent advances in understanding the biology, epidemiology and control of chlamydial infections in koalas. Veterinary Microbiology.

[11] Wilson DP, Craig AP, Hanger J, Timms P (2015). The paradox of euthanizing koalas (*Phascolarctos cinereus*) to save populations from elimination. Journal of Wildlife Diseases.

[12] Rhodes JR (2011). Using integrated population modelling to quantify the implications of multiple threatening processes for a rapidly declining population. Biological Conservation.

[13] Perry LL, Feilzer K, Caldwell HD (1997). Immunity to *Chlamydia trachomatis* is mediated by T helper 1 cells through IFN-gamma-dependent and -independent pathways. The Journal of Immunology.

[14] Holland M (1996). T helper type‐1 (Th1)/Th2 profiles of peripheral blood mononuclear cells (PBMC); responses to antigens of *Chlamydia trachomatis* in subjects with severe trachomatous scarring. Clinical & Experimental Immunology.

[15] Yang X, Gartner J, Zhu L, Wang S, Brunham RC (1999). IL-10 gene knockout mice show enhanced Th1-like protective immunity and absent granuloma formation following Chlamydia trachomatis lung infection. The Journal of Immunology.

[16] Menon S (2015). Human and Pathogen Factors Associated with Chlamydia trachomatis-Related Infertility in Women. Clinical Microbiology Reviews.

[17] Natividad A (2008). Susceptibility to sequelae of human ocular chlamydial infection associated with allelic variation in IL10 cis-regulation. Human Molecular Genetics.

[18] Kinnunen AH (2002). HLA DQ alleles and interleukin-10 polymorphism associated with Chlamydia trachomatis-related tubal factor infertility: a case–control study. Human Reproduction.

[19] Andrew DW (2013). The duration of *Chlamydia muridarum* genital tract infection and associated chronic pathological changes are reduced in IL-17 knockout mice but protection is not increased further by immunization. PLoS ONE.

[20] Mathew M, Waugh C, Beagley KW, Timms P, Polkinghorne A (2014). Interleukin 17A is an immune marker for chlamydial disease severity and pathogenesis in the koala (*Phascolarctos cinereus*). Developmental & Comparative Immunology.

[21] Fonfara S, Siebert U, Prange A, Colijn F (2007). The impact of stress on cytokine and haptoglobin mRNA expression in blood samples from harbour porpoises (*Phocoena phocoena*). Journal of the Marine Biological Association of the United Kingdom.

[22] Erdèlyi K (2005). Gallotannin inhibits the expression of chemokines and inflammatory cytokines in A549 cells. Molecular Pharmacology.

[23] Chen L, Zhao L, Zhang C, Lan Z (2014). Protective effect of p-cymene on lipopolysaccharide-induced acute lung injury in mice. Inflammation.

[24] Nelson RJ (2004). Seasonal immune function and sickness responses. Trends in Immunology.

[25] Acevedo-Whitehouse K, Cunningham AA (2006). Is MHC enough for understanding wildlife immunogenetics?. Trends in Ecology & Evolution.

[26] Lau Q, Griffith JE, Higgins DP (2014). Identification of MHCII variants associated with chlamydial disease in the koala (Phascolarctos cinereus). PeerJ.

[27] Muehlenbein MP, Bribiescas RG (2005). Testosterone‐mediated immune functions and male life histories. American Journal of Human Biology.

[28] Nelson RJ, Demas GE, Klein SL, Kriegsfeld LJ (1995). Minireview: The influence of season, photoperiod, and pineal melatonin on immune function. Journal of Pineal Research.

[29] McInnes LM (2009). Trypanosoma irwinz n. sp (Sarcomastigophora: Trypanosomatidae) from the koala (Phascolarctos cinereus). Parasitology.

[30] McInnes L, Hanger J, Simmons G, Reid S, Ryan U (2011). Novel trypanosome Trypanosoma gilletti sp.(Euglenozoa: Trypanosomatidae) and the extension of the host range of Trypanosoma copemani to include the koala (Phascolarctos cinereus). Parasitology.

[31] McInnes L, Gillett A, Hanger J, Reid S, Ryan U (2011). The potential impact of native Australian trypanosome infections on the health of koalas (Phascolarctos cinereus). Parasitology-Cambridge.

[32] Barbosa A (2016). First report of Trypanosoma vegrandis in koalas (Phascolarctos cinereus). Parasitology International.

[33] Vaz P (2011). Detection of a Novel Gammaherpesvirus in Koalas (Phascolarctos cinereus). Journal of Wildlife Diseases.

[34] Vaz P (2012). Detection of a Second Novel Gammaherpesvirus in a Free-ranging Koala (Phascolarctos cinereus). Journal of Wildlife Diseases.

[35] Maher IE, Higgins DP (2016). Altered Immune Cytokine Expression Associated with KoRV B Infection and Season in Captive Koalas. PLoS ONE.

[36] Margolick JB (2006). Impact of inversion of the CD4/CD8 ratio on the natural history of HIV-1 infection. Journal of Acquired Immune Deficiency Syndromes.

[37] Taylor JM, Fahey JL, Detels R, Giorgi JV (1989). CD4 percentage, CD4 number, and CD4: CD8 ratio in HIV infection: which to choose and how to use. Journal of Acquired Immune Deficiency Syndromes.

[38] Hartmann K (2012). Clinical aspects of feline retroviruses: a review. Viruses.

[39] Hofmann-Lehmann R, Holznagel E, Ossent P, Lutz H (1997). Parameters of disease progression in long-term experimental feline retrovirus (feline immunodeficiency virus and feline leukemia virus) infections: hematology, clinical chemistry, and lymphocyte subsets. Clinical and Diagnostic Laboratory Immunology.

[40] Quackenbush SL (1990). Lymphocyte subset alterations and viral determinants of immunodeficiency disease induction by the feline leukemia virus FeLV-FAIDS. Journal of Virology.

[41] Tompkins M, Nelson P, English R, Novotney C (1991). Early events in the immunopathogenesis of feline retrovirus infections. Journal of the American Veterinary Medical Association.

[42] Pedersen AB, Babayan SA (2011). Wild immunology. Molecular Ecology.

[43] Beineke A, Siebert U, Van Elk N, Baumgärtner W (2004). Development of a lymphocyte-transformation-assay for peripheral blood lymphocytes of the harbor porpoise and detection of cytokines using the reverse-transcription polymerase chain reaction. Veterinary Immunology and Immunopathology.

[44] Landolfi JA (2010). Comparison of systemic cytokine levels in Mycobacterium spp. seropositive and seronegative Asian elephants (Elephas maximus). Journal of Zoo and Wildlife Medicine.

[45] Mangar C, Armitage CW, Timms P, Corcoran LM, Beagley KW (2016). Characterisation of CD4 T cells in healthy and diseased koalas (Phascolarctos cinereus) using cell-type-specific monoclonal antibodies. Developmental & Comparative Immunology.

[46] Jayarapu K, Kerr MS, Katschke A, Johnson RM (2009). Chlamydia muridarum-specific CD4 T-cell clones recognize infected reproductive tract epithelial cells in an interferon-dependent fashion. Infection and Immunity.

[47] Clerici M, Shearer GM (1994). The Th1–Th2 hypothesis of HIV infection: new insights. Immunology Today.

[48] Clerici M, Shearer GM (1993). A Th 1→ Th 2 switch is a critical step in the etiology of HIV infection. Immunology Today.

[49] Linenberger ML, Deng T (1999). The effects of feline retroviruses on cytokine expression. Veterinary Immunology and Immunopathology.

[50] Graham EM, Jarrett O, Flynn JN (2003). Development of antibodies to feline IFN-γ as tools to elucidate the cellular immune responses to FeLV. Journal of Immunological Methods.

[51] Tompkins MB, Tompkins WA (2008). Lentivirus-induced immune dysregulation. Veterinary Immunology and Immunopathology.

[52] Pickup JC, Chusney GD, Thomas SM, Burt D (2000). Plasma interleukin-6, tumour necrosis factor α and blood cytokine production in type 2 diabetes. Life Sciences.

[53] Morrison SG, Su H, Caldwell HD, Morrison RP (2000). Immunity to murine Chlamydia trachomatis genital tract reinfection involves B cells and CD4+ T cells but not CD8+ T cells. Infection and Immunity.

[54] Beatty WL, Belanger TA, Desai AA, Morrison RP, Byrne GI (1994). Tryptophan depletion as a mechanism of gamma interferon-mediated chlamydial persistence. Infection and Immunity.

[55] Igietseme JU (1998). Route of infection that induces a high intensity of gamma interferon-secreting T cells in the genital tract produces optimal protection against Chlamydia trachomatis infection in mice. Infection and Immunity.

[56] Natividad A (2005). Risk of trachomatous scarring and trichiasis in Gambians varies with SNP haplotypes at the interferon-gamma and interleukin-10 loci. Genes and Immunity.

[57] Speight KN (2016). Prevalence and pathologic features of *Chlamydia pecorum* infections in South Australian koalas (*Phascolarctos cinereus*). Journal of Wildlife Diseases.

[58] Legione AR (2016). Identification of unusual Chlamydia pecorum genotypes in Victorian koalas (Phascolarctos cinereus) and clinical variables associated with infection. Journal of Medical Microbiology.

[59] Patterson JL (2015). The prevalence and clinical significance of Chlamydia infection in island and mainland populations of Victorian koalas (*Phascolarctos cinereus*). Journal of Wildlife Diseases.

[60] Martin, R. W. & Handasyde, K. A. The koala: natural history, conservation and management. (University of New South Wales Press 1999).

[61] Malkin CJ (2004). The effect of testosterone replacement on endogenous inflammatory cytokines and lipid profiles in hypogonadal men. The Journal of Clinical Endocrinology & Metabolism.

[62] Zhang Y-Z, Xing X-W, He B, Wang L-X (2007). Effects of testosterone on cytokines and left ventricular remodeling following heart failure. Cellular Physiology and Biochemistry.

[63] Li Z, Yue Y, Xiong S (2013). Distinct Th17 inductions contribute to the gender bias in CVB3-induced myocarditis. Cardiovascular Pathology.

[64] McLean, N. *Ecology and management of overabundant koala (Phascolarctos cinereus) populations* PhD thesis, University of Melbourne, (2003).

[65] Tarlinton R, Meers J, Hanger J, Young P (2005). Real-time reverse transcriptase PCR for the endogenous koala retrovirus reveals an association between plasma viral load and neoplastic disease in koalas. Journal of General Virology.

[66] Maher IE, Griffith JE, Lau Q, Reeves T, Higgins DP (2014). Expression profiles of the immune genes CD4, CD8β, IFNγ, IL-4, IL-6 and IL-10 in mitogen-stimulated koala lymphocytes (*Phascolarctos cinereus*) by qRT-PCR. PeerJ.

[67] Schmittgen TD, Livak KJ (2008). Analyzing real-time PCR data by the comparative CT method. Nature Protocols.

